# Are Students Really Cautious about Food Waste? Korean Students’ Perception and Understanding of Food Waste

**DOI:** 10.3390/foods9040410

**Published:** 2020-04-02

**Authors:** Maidul Islam

**Affiliations:** Department of E-Trade, College of Social Science, Keimyung University, Dalseo-gu, Daegu 42601, Korea; maidul@kmu.ac.kr; Tel.: +82-53-580-5967

**Keywords:** food waste, students’ perception, household food, purchase behavior, exogenous effects

## Abstract

The amount of food wasted by Korean households is significant and to some extent could be preventable. It is not well illustrated how Korean students perceive food waste and how much they know about the consequences of food waste. This study aimed to examine Korean students’ perception of food waste. Overall, results show that students’ perception of food waste varies in different clusters. Considerate food wasters (cluster 1) are knowledgeable and have much information regarding food waste. This paper suggests that additional information about how to preserve food and about issues related to food wastes, which cause a bigger environmental problem over the long term, could influence the behavior of this cluster, reducing perhaps further food waste. On the other hand, unwitting and ruthless food wasters, who are in clusters 2 and 3, need extra attention. Marketers should initiate educational campaigns to raise awareness of food waste for students and youth. Students who fall under these two clusters may need to pay extra attention to their shopping behavior. They should be more connected to their food, and to their purchase behavior, which may reduce food waste.

## 1. Introduction

### Background and Objectives

Food waste is simply food intended for consumption that is discarded along the food supply chain or the process of cooking, agriculture, or livestock production. According to Parfitt, Barthel, and Macnaughton [1], food waste is all the food that is available but not eaten and wasted. Food waste is an international issue that is presently gaining extra attention due to food security problems and other environmental problems. Gustavsson, Cederberg, Sonesson, Otterdijk, and Meybeck [2] have explained in their research work that one-third of edible food is wasted every year. Much of this waste comes from high-income nations due to their poor marketing practices and purchasing behavior [3]. Earlier research has explained that there is a need for systematic change among the actors of the food system’s food chain [4]. Several other studies have explored food waste as a result of the irresponsible behavior of society. Decreasing the amount of food waste is an important factor for a sustainable environment. In fact, as per Graham-Rowe, Donna, Jessop, and Sparks as well as Stuart [3,5], the waste of food is a financial loss and this loss indirectly makes food less accessible to the poorest people. According to 2017 data from Korea’s Ministry of Environment (Land & Waste) [6], individual Korean citizens discharged 1.02 kg of household food waste daily, which is one-third of the amount discharged in 1991. Furthermore, the website also mentioned that food waste is decreasing every year in Korea; in 2014 it was 4,139,083 tons and in 2015 it came down to 3,821,594 tons, followed by 3,845,124 tons in 2016 and 3,430,622 ton in 2017. These data certainly give a positive impression and each country should adopt a similar mechanism to reduce waste; however, the difficulty in following such a path is the cost involved. The cost of processing food waste has been increasing due to an increase in transportation, labor, material, and power costs, and other external factors. Food waste disposal costs are skyrocketing every year. Food waste costs around 110,000 won (approximately $100 per ton, including materials, labor, power, and water [7]. Looking at the amount of money involved in food waste management, it is clear that everyone should pay attention to their food waste. Specifically, young adult college and university students, who have proven to be more wasteful [8], should play an important role in food waste management. Hence, this paper aimed to find out about students’ perception, awareness, and amount of information they have about food waste. The students were grouped into three different clusters based on their perception, awareness, and degree of information about food waste. 

## 2. Literature Review—Waste of Food in Households and Educational Institute 

Food waste is the disposal of potentially usable food. Countries are trying to reduce food waste because it is both wasteful and expensive to dispose of. The causes of food waste are increasing due to higher quality of living standards [9]. Food waste is said to occur at all stages of the food chain, that is, 28% of waste occurs at the consumer level, another 28% at the production level, and the remaining percentage gets lost at the distribution, storage as well as processing levels [2]. The disposal of food waste also plays an important role since it includes high costs and complex processes that are followed so no one can say that it has any impact on the environment. Food waste consists of all kinds of leftovers, which can be either unavoidable or partly avoidable, or even avoidable. To avoid food waste, a volume-based fee system has been implemented for the residents of South Korea. However, the amount of food waste is increasing due to the improvement in people’s living standards. As per the Korean official site [6], the majority of household waste is account for by single or two-people households, being around 48%. This prompts the conclusion that there is a clear need to reduce food waste. A similar conclusion was drawn by Brook [8] in that single-person households or young professionals often buy more and eat less. In 2012, the daily generated amount of food waste was about 13,209 tons, which makes up about 27% of the total amount of generated residential waste (48,990 tons/day) [6]. If the citizens of this country do not pay enough attention to food waste, it is likely that food waste will grow gradually as people’s living standards are improving year after year. 

According to the Korean Ministry of Environment, 70% of food waste comes from homes and small restaurants. With the recent increase in the number of people living alone, the amount of food waste is growing exponentially. Food waste causes environmental pollution and much economic damage during the process. As per the World Economic Forum, a Korean family with six people creates approximately 780 kg of food waste each year [10], which requires at least 148 trees to absorb this in a year. If people managed to waste 20% less than what they are now wasting, this could reduce waste disposal costs by 160 billion won per year and save energy by 5 trillion won [6]. 

Korea ranks above the average in housing, education, and earning power [11]. With the rapid economic development, Korea has changed and diversified, accompanied by improved living standards [12]. In particular, as the number of occasions to eat out has increased, the amount of food waste has increased. Food waste in the diet is causing serious economic damage and environmental problems. According to the Ministry of Environment’s press release, South Korea has strengthened its food waste system and improved its legal system to expand recycling. However, food waste is still not being properly reduced and is causing serious environmental problems. The Korean consumer considers the issue of food waste predominantly as a social problem and less as an environmental or economic problem. In other words, Korean consumers barely see a relation between food waste and environmental problems. Moreover, food waste can cause negative emotions; thus, wasteful consumption is associated with guilt and many consumers have a bad conscience about wasting food. Many consumers know how they can reduce their food waste at home, but do not act according to recommendations. Supplying family members and guests with abundant and healthy food is often a reason for the development of food waste. Furthermore, it is difficult to convince all household members to change their behavior and reduce food waste (Korea Environment Corporation). University cafeterias and school canteens are considered places where food waste can be noted significantly, and these are mostly run by public bodies [13,14]. Managing the school meal waste issue could resolve several environmental problems [14,15,16]. Garcia-herrero, L Menna, and Vittuari [17] suggested that an improvement in food consumption and waste at school should be studied as it could resolve food waste-related problems. Earlier research [18] has also suggested that around 78% of respondents believed that students engaging more in socializing than in focusing on eating was the reason for food waste on the school campus. The authors further suggested that younger elementary school students wasted more food than older ones [18].

## 3. Materials and Methods

### 3.1. Methodical Approach

An online survey was conducted to collect sample for this research work. The participants were randomly selected from students who were studying at Korean universities. The online survey was conducted from 3 to 20 May 2019. The survey was designed to obtain insights into Korean students’ behavior and perceptions of food waste and to classify them into different groups or clusters according to their responses. The idea of food waste was clearly explained to the respondents at the initial stage of the survey, so they clearly understand what waste was and was not. In all, 439 students took part in the survey from different universities in South Korea. The questions included socio-demographic aspects as well as aspects of purchasing behavior and one’s own assessment of food waste. Most of the questions or statements had to be answered on a scale of 1 (do not agree at all) to 5 (absolutely agree). The questionnaire was adopted from an earlier research paper presented by Richter [19], with a slight modification based on this research requirement.

An exploratory factor analysis was performed to reduce the data to a smaller set of summary variables. The final cluster analysis aimed to identify different groups with regard to their behavior and perception of food and food waste within the sample. The variation between the groups should be as heterogeneous as possible and as homogeneous as possible within the group [20]. In keeping with [19], the cluster analysis was executed in several steps. In the first step, the nearest neighbor method was applied to eliminate outliers. In the second step, Ward’s method was used to determine the number of clusters and the average values of the clusters. Then, using k-means, the validity of the cluster solution was confirmed [20]. The discriminatory analysis was done to test group assignments. Finally, Tamhane’s significant differences between and within clusters were verified.

### 3.2. Sample Description

A total of 439 students participated in this research study. However, out of the total, a few (6) were international exchange students and were excluded from the main research and a few (4) submitted incomplete questionnaires. Therefore, a total of 429 samples were used for this research (Table 1). The survey participants were in the age group of 19 to 28 years old. More than 50% of the students were in the age group of 22–24 years. Furthermore, the sample consisted of 55.7% females and 44.3% males. The majority of the participants had an income of 200,000–400,000 won; however, their average monthly expenses were 560,000 won, which is higher than their income because students’ expenses are mostly supported by their parents. Participants lived mostly with parents (49.6%), followed by a shared flat (34.7%) and a dormitory (12.5%). The study cannot be considered representative of Korea as a whole since most students came from the Daegu area, which is located in the southern part of Korea, as is mentioned in the Conclusions section. However, Daegu is home to more than 2 million people and the collected sample provides a clear picture of students’ behavior and perception about food waste.

## 4. Results

### 4.1. Reliability Analysis

The first part of the analysis consisted of checking internal consistency using the key figure, Cronbach’s alpha (α). The higher the α coefficient, the more the elements share the covariance and the higher the probability that they measure the same underlying concept. An α coefficient between 0.65 and 0.8 (or higher) is recommended by many methodologists. Coefficients less than 0.5 are generally unacceptable. The item question “Influence on expiration date” and “Food waste in households” could not be considered for further analysis due to insufficient coefficients. In addition, the item “The food I waste would not help undernourished people” from factor 2 and the item “use food waste arising from food preparation often otherwise, for example, boiling of bones” from factor 4 were eliminated to increase internal consistency.

### 4.2. Factor Analysis

Factor analysis is a method of grouping similar variables or item questions into dimensions. This process is used to identify latent variables or contracts. The overall goal of factor analysis is to reduce many individual items into a smaller number of dimensions. The correlation coefficient “factor loading” measures the relationship between a symptom and a factor. Structures in large quantities of variables can be identified and relationships between a large number of variables can be structured by the identification of variable groups (factors). For the factor analysis, the rotated component matrix was used to identify the variable groups (factors). Based on the results of the rotated component matrix, four factors were identified (Table 2).

The first factor (emotions) aggregates statements about the negative feelings when wasting food (e.g., feelings of guilt). The second factor (environment) includes allegations of environmental damage caused by food waste. Statements on the purchasing behavior of the participants are summarized in factor number three. With factor number four (Handling food), the behavior during and after cooking with food is explored. The factors identified were used for clustering to highlight issues that could be used to inform students about how to minimize food waste. Table 2 gives an overview of the results of the reliability analysis (Cronbach´s alpha) and the factor analysis (factor loading).

### 4.3. Cluster Analysis

**Figure 1:**
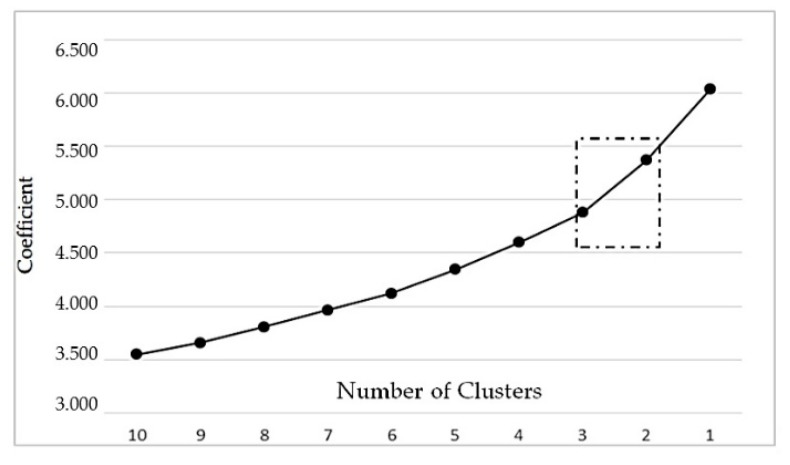
Analysis of the coefficients from the agglomeration schedule.

**Figure 2:**
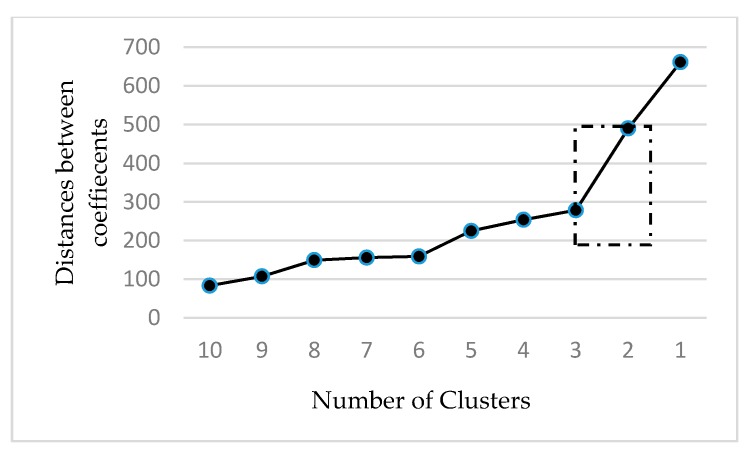
Analysis of the distances between the coefficients from the agglomeration schedule.

The cluster analysis was executed in several steps. In the first step, the nearest neighbor method (single-linkage method) was applied to eliminate outliers. The squared Euclidean distance was chosen as the measurement interval. In this regard, eleven outliers were identified and removed from the data set. Then, Ward’s method was executed to create groups that obtained the highest heterogeneity between groups and the lowest homogeneity within groups [21]. In this context, the agglomeration schedule was used to identify the highest increase of heterogeneity when creating or removing an additional cluster. For this purpose, an analysis of the coefficients from the agglomeration schedule and the distances between the coefficients was executed. In this regard, line diagrams added in the appendix were used. It is especially noticeable in the line diagram with the distances of the coefficients that a three-cluster solution is appropriate, because at this point (decrease from 3 to 2 clusters) the highest slope can be observed (Figure 1 and Figure 2). In order to maintain an appropriate heterogeneity between the groups, this group formation makes the most sense.

To check statistical significance, the results from a one-way ANOVA analysis are used. A significant result for single-factor ANOVA means that at least two groups differ statistically significantly from each other. In summary, it can be said that each factor differs statistically at least significantly from another factor. This is shown in the last column of the ANOVA analysis, which shows a significance of 0.000 in each factor (Table 3).

Three clusters in particular show differences in factors one (Individual effects), two (Exogenous effects), and three (Purchase behavior).These factors were especially taken into account when naming the clusters: Considerate food wasters (1), unwitting food wasters (2), and ruthless food wasters (3). The clusters and the average values of the individual items and factors are presented in Table 4.

In comparison with clusters 1 and 3, considerate food wasters have the highest agreement with the statements about individual effects (µ = 3.29) and handling food (µ = 4.09). However, surprisingly, cluster 1 shows the least agreement with the statements about exogenous effects (µ = 1.94). This may be because of their lesser knowledge about the consequences of food waste or they may think they waste a very negligible amount of food; hence, it would not affect the environment. This cluster contains 206 participants and is the largest cluster.

Cluster 2 (unwitting food wasters) has high agreement with the statement of purchase behavior (µ = 3.58) and exogenous effects (µ = 3.12). However, it shows low concern about individual effects (µ = 2.95). Cluster 2 has 166 students, which is around 40% of the collected sample.

Cluster 3 (ruthless food wasters) has the least agreement with the statements from factor 1 (individual effects; µ = 1.76), factor 3 (purchase behavior; µ = 1.98), and also factor 4 (handling food; µ = 3.26) compared to the other two clusters. On the other hand, it agrees most with the statements from factor 2 (exogenous effects; µ = 3.14). It contains 46 students and is the smallest cluster.

## 5. Discussion

Culturally, South Korea is different from other countries and its food culture is unique. Student cafeterias in South Korea serve several types of side dishes that mostly get wasted as students can only manage to eat a limited amount of them. Hence, it is important to look into students’ perception of food waste in South Korea. The aim of this study was to show the perception differences about food waste between three groups, which were classified as considerate food wasters (cluster 1), unwitting food wasters (cluster 2), and ruthless food wasters (cluster 3). Results of this study suggested that considerate food wasters, who are in cluster 1, consider food waste a serious issue and would like to get more knowledge about it. Providing further information could help them to reduce food waste even further. On the other hand, results about clusters 2 and 3 showed less concern about leftover food. To be more specific, cluster 3 need to be more sensitized about food waste. Earlier research carried out by Cox and Downing [22] showed that excessive cooking or purchasing leads to food waste. A similar conclusion was drawn by Williams, Wikstrom, Otterbring, Lofgren, and Gustafsson [23], as well as by Koivupuro, Hartikainen, Silvennoinen, Katajajuuri, Heikintalo, Reinikainen, and Jalkanen [24]. Hence, it is clear that a lack of proper planning on purchasing and cooking leads to food waste. Previous research done by Hamilton, Denniss, and Baker [25] explained that customers often have a sense of wrongdoing when they waste food. Other research studies also explored the concept that customers feel uncomfortable when they waste food [3,26]. Hence, this research also suggested that having an intense campaign to make consumers feel guilty about food waste can bring awareness within the buyers and can lead to a reduction in food waste.

Results from Table 5 show that cluster 1 has more females than the other clusters and that gender could be considered to have some effect on food waste. Besides gender, the age group may also play some role in food waste. College students are more exposed to Internet ads as well as television ads and are highly likely to consume ready-made meals at home, which results in more food waste [27]. Marketers need to be more socially responsible when promoting their products and motivate college students not to waste food. Marketers should find ways to encourage students to reduce the amount of leftover food as they are more likely to have an uncontrollable buying behavior.

Like other research papers, this paper also has several limitations. First, the samples were mostly taken from the universities located in the Daegu area. Samples taken from all over the country and bigger sample sizes could give us a better insight into the student population. Furthermore, the samples were undergraduate students; adding Master’s and Ph.D. students could also produce more comprehensive results.

Data from cluster 1 showed more females than in the other clusters. Participants in this cluster are comparatively older than those in clusters 2 and 3. This shows that senior students are comparatively more responsible than junior students. Research carried out by Hamilton et al. [25] showed that older people are more responsible when it comes to food waste. Earlier purchasing behavior may have influenced their buying decision and food waste attitude [28].

In a common scenario, people think that they do not waste much and that a small portion of waste can be acceptable [29]. These food wasters do not consider this as a problem and do not know the ecological consequences of the waste; hence, they feel it is socially acceptable. Therefore, it is essential to let them know that the problem arises due to that waste.

## 6. Conclusions

This study aimed to examine Korean students’ perception towards food waste. Overall, results show that students’ perception toward food waste varies in different clusters. Considerate food wasters (cluster 1) are knowledgeable and have much information regarding food waste. It was asserted that additional information could influence the behavior of this cluster, reducing perhaps further food waste. On the other hand, unwitting and ruthless food wasters, who are in clusters 2 and 3, need extra attention. These two clusters may need to be more connected to their food and should purchase things intelligently and realistically. They should be informed that, when cooking, they should not over-serve or cook excessively. Moreover, they should also be informed about how to store food correctly, for example, vegetables like potatoes and onions should not be refrigerated. Extra information about the adverse effects of food waste should be provided to clusters 2 and 3. They should be informed that a significant source of methane (greenhouse gas) is food waste.

Finally, this paper helps us to understand that students are not well informed about the consequences of food waste. Further study is needed to find a better way to make students understand about food waste.

## Figures and Tables

**Table 1:** Summary of the socio-demographic data about the participating students.

Variable	Description	Frequency (%) Sample
Age	19–21	26.8%
	22–24	53.15%
	25–28	20.05%
Gender	Female	55.7%
	Male	44.3%
Monthly Expenses	0–200,000 won	5.83%
	200,001–400,000 won	47.09%
	401,000–600,000 won	27.97%
	600,001–1,000,000 won	15.38%
	1,000,001 won or more	3.73%
Living Situation	Live alone in an apartment	3.03%
	Live at home with parents	49.65%
	Live in a shared flat	34.73%
	Live in a dormitory	12.59%

**Table 2:** Results of the reliability and factor analyses.

	Reliability Analysis and Factor Analysis	
	Reliability Analysis	Factor Loading
Factor 1: Individual effects (emotions) i. I have a bad conscience when I waste food.	0.892	0.922
ii. I feel guilty when I waste food.		0.927
Factor 2: Exogenous effects (environment) i. I don´t care about environmental impairments which arise when I discard food.	0.733	0.733
ii. I don’t care about effects of food waste regarding the global resource availability.		0.850
iii. Food waste is not an environmental problem, because it is natural and biodegradable.		0.786
Factor 3: Purchase behavior i. Usually, I seriously consider what I will buy before purchasing.	0.748	0.584
ii. I only buy products which are on my shopping list.		0.847
iii. I plan meals for several days to purchase more efficiently.		0.756
Factor 4: Handling food i. During food preparation I take care to use everything possible.	0.795	0.664
ii. If something remains after cooking, I freeze it for later use.		0.868
iii. I eat food leftovers the next day.		0.769

**Table 3:** ANOVA analysis presenting item questions and their level of significance. IE, Individual Effects; EXE, Exogenous effects; PB, Purchase Behavior; HF, Handling Food; df, Degree of Freedom; F, f-value; Sig.,Significant.

Code	Item Questions		Sum of Squares	df	Mean Square	F	Sig.
IE(1)	I have a bad conscience when I waste food.	Between Groups	100.182	2	50.091	45.140	0.000
		Within Groups	460.519	415	1.1110		
		Total	560.701	417			
IE(2)	I feel guilty when I waste food.	Between Groups	77.007	2	38.504	34.292	0.000
		Within Groups	465.971	415	1.123		
		Total	542.978	417			
EXE(1)	I don’t care about environmental impairments which arise when I discard food.	Between Groups	146.043	2	73.022	71.988	0.000
		Within Groups	420.961	415	1.014		
		Total	567.005	417			
EXE(2)	I don’t care about effects of food waste regarding the global resource availability.	Between Groups	121.588	2	60.794	59.276	0.000
		Within Groups	425.627	415	1.026		
		Total	547.215	417			
EXE(3)	Food waste is not an environmental problem, as it is natural and biodegradable.	Between Groups	179.532	2	89.766	92.522	0.000
		Within Groups	402.640	415	0.970		
		Total	582.172	417			
PB(1)	Normally, I seriously consider what I will buy before purchasing.	Between Groups	56.806	2	28.403	32.872	0.000
		Within Groups	358.581	415	0.864		
		Total	415.388	417			
PB(2)	I only buy products which are on my shopping list.	Between Groups	157.899	2	78.950	88.261	0.000
		Within Groups	371.220	415	0.895		
		Total	529.120	417			
PB(3)	I plan meals for several days to purchase more efficiently.	Between Groups	137.151	2	68.576	63.744	0.000
		Within Groups	446.459	415	1.076		
		Total	583.610	417			
HF(1)	During food preparation I take care of using everything possible.	Between Groups	83.632	2	41.816	48.092	0.000
		Within Groups	360.846	415	0.870		
		Total	444.478	417			
HF(2)	If something remains after cooking, I freeze it for later use.	Between Groups	32.496	2	16.248	23.277	0.000
		Within Groups	289.677	415	0.698		
		Total	322.172	417			
HF(3)	I eat food leftovers the next day.	Between Groups	48.995	2	24.497	26.931	0.000
		Within Groups	377.493	415	0.910		
		Total	426.488	417			

**Table 4:** Result of the cluster analysis—cluster forming factors.

Final Cluster Centers			
	Cluster 1	Cluster 2	Cluster 3
	Considerate Food Wasters	Unwitting Food Wasters	Ruthless Food Wasters
Cluster size, absolute number and in percentage (%)	206 (48.02%)	166 (39.7%)	46 (11%)
Factor 1: Individual effects (emotions)	3.29	2.95	1.76
IE(1). I have a bad conscience when I waste food	3.41	3.02	1.78
IE(2). I feel guilty when I waste food.	3.17	2.89	1.74
Factor 2: exogenous effects (environment)	1.94	3.12	3.14
EXE(1). I don’t care about environmental impairments which arise when I discard food.	2.05	3.19	3.37
EXE(2). I don’t care about effects of food waste regarding the global resource availability.	2.07	3.18	3.00
EXE(3). Food waste is not an environmental problem, because it is natural and biodegradable.	1.69	2.99	3.04
Factor 3: Purchase behavior	3.06	3.58	1.98
PB(1). Usually I seriously consider what I will buy before purchasing.	4.05	3.90	2.83
PB(2). I only buy products which are on my shopping list.	2.47	3.42	1.54
PB(3). I plan meals for several days to purchase more efficiently.	2.65	3.41	1.56
Factor 4: Handling food	4.09	3.75	3.26
HWF(1). During food preparation I take care to use everything possible.	3.75	3.75	2.33
HWF(2). If something remains after cooking, I freeze it for a later use.	4.25	4.04	3.31
HWF(3). I eat food leftovers the next day.	4.21	3.63	3.30

**Table 5:** Age and gender based on clusters.

	Cluster 1 Considerate Food Wasters	Cluster 2 Unwitting Food Wasters	Cluster 3 Ruthless Food Wasters
Age	19–21 years: 16.9%	19–21 years: 23.2%	19–21 years: 31.8%
	22–24 years: 19.4%	22–24 years: 44.6%	22–24 years: 39.3%
	25–28 years: 63.7%	25–28 years: 32.2%	25–28 years: 28.9%
Gender	Male: 41.1%	Male: 47.4%	Male: 53.3%
	Female: 58.9%	Female: 52.6%	Female: 46.7%
